# Supplementary material for: Direct Capture and Early Detection of Lyme Disease Spirochete in Skin with a Microneedle Patch
Source: Biosensors (Basel). 2022 Oct 2;12(10):819. doi: 10.3390/bios12100819 (PMC9599122; doi:10.3390/bios12100819)
Supplement: Supplementary file 1 [file biosensors-12-00819-s001.zip › biosensors-1920415-supplementary.pdf]

Supplemental Materials

Direct Capture and Early Detection of Lyme Disease Spirochete in Skin with a Microneedle Patch

Emily Kight [1], Rosana Alfaro[1], Shiva Kumar Goud Gadila[2], Shuang Chang[1], David Evans[1], Monica Embers[2] and Frederick Haselton[1,*]

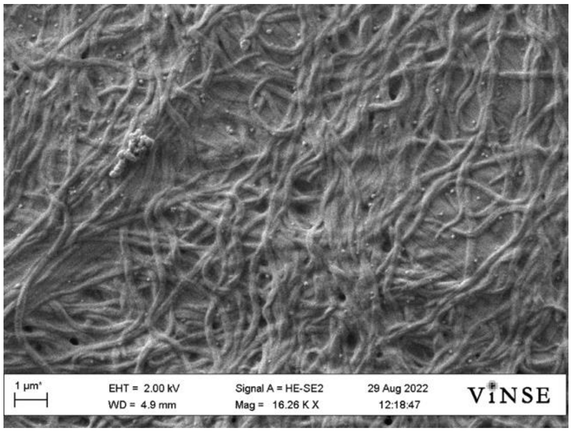

Figure S1. SEM of *B. Burgdorferi* on parafilm.

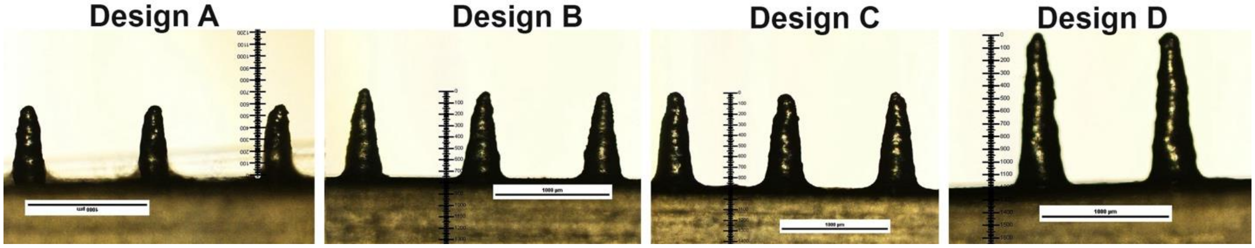

Figure S2. 3D printed MN designs shown in table 1 imaged on light microscope (2x).

Type of PVA	Average Viscosity (cP)
PVA M_w 13-23K	15.7 ± 2.5
**PVA M_w 30-50K**	**48.4 ± 6.1**
PVA M_w 85-124K	377.6 ± 2.8

Table S1. Average Viscosity measurements of different molecular weight PVA. The same solutions were measured three times each and average ± SD.

Type	13-23K (g)	31-50K (g)	85-124K (g)
average 50 ul droplet	0.0451 ± 0.002	0.0465 ± 0.005	0.0266 ± 0.007

Table S2. Pipette test of different molecular weight PVA. 50 μl of each solution was pipetted onto a weigh boat three times. Average ± SD.

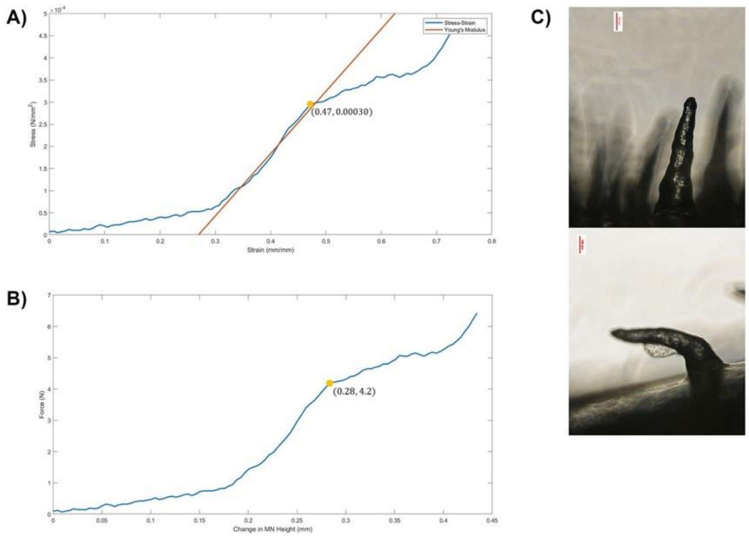

Figure S3. Representative compression test results. A) shows the stress-strain curve, B) shows the deformation force. C) shows light microscope pictures before and after 80K g force was applied to a MN (scale bar 100 μm).

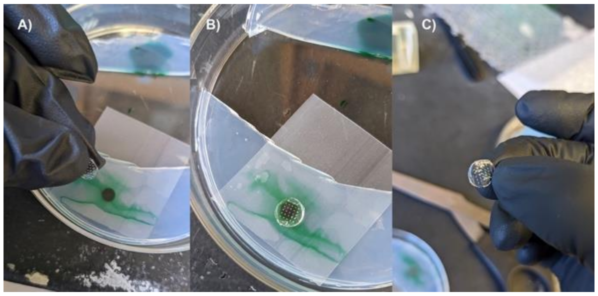

Figure S4. Qualitative absorption test. A) A sheet of PF is placed above a 2%(w/w) gel with 10 μl of green dye in a hole in the gel. B) A MN is applied to the PF and gel. C) A MN is removed after 10 minutes and needles from the patch show the green dye is absorbed.

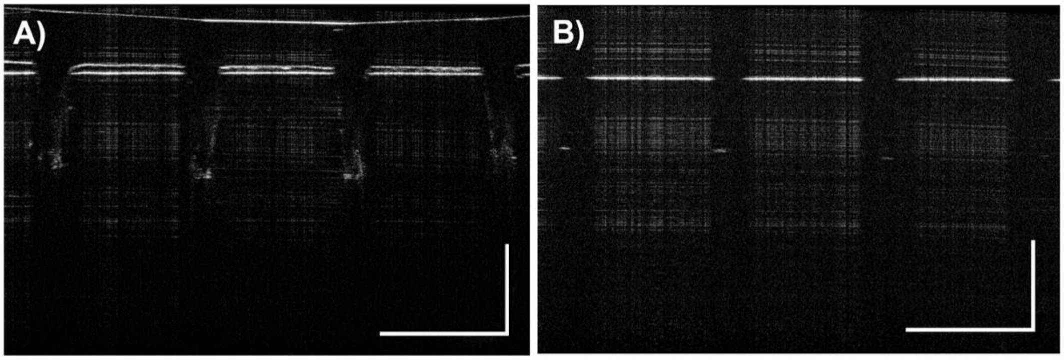

Figure S5. OCT 2D cross-sectional (B-scan) images visualization of holes in 2% (w/w) gel from MN. A) 2% (w/w) gel with a MN inserted on top. B) After MN removal, holes are in gel (space between horizontal white lines). Scale bar 1000 μm.

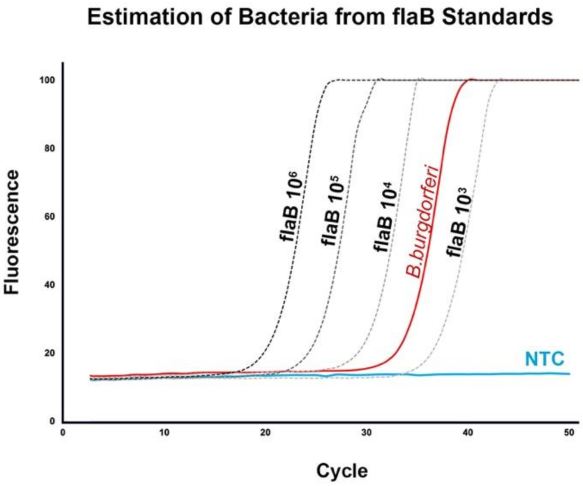

Figure S6. The estimation of *B. burgdorferi* (red) based on flaB standard curve (black).

To confirm the number of bacteria after a possible loss during reconstitution and possible degradation of bacteria in tubes, a tube of bacteria was tested in the PCR. The standard curve shows intensity of fluorescence versus cycle number with the separation of curves in proportion to the logarithmic dilutions of target (*B. burgdorferi* gene flaB). The limit of detection of PCR reagents was found to be 180 copies per reaction. The R^2 of the standard curve was found to be 0.998 (**Figure S5**).

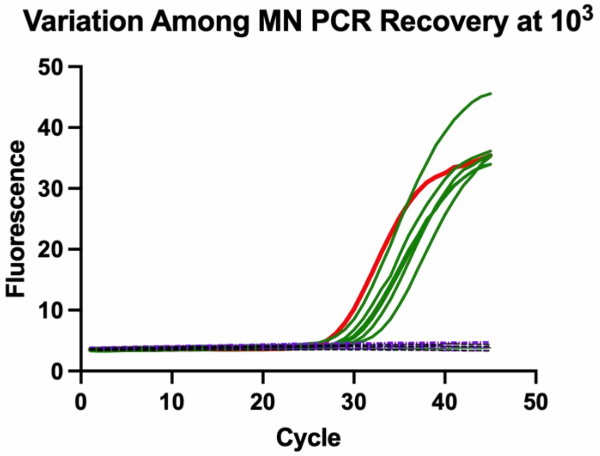

Figure S7. Single PCR experiment with 4 pig MN extractions spiked with 10^3 bacteria. Each PCR was run in duplicate. The positive patches are shown in green. The red line shows the spiked positive control. The dashed black lines are the NTC tubes and the dashed purple lines are the negative MN patches.
